# Musculoskeletal disorders among older hospital cleaners: a systematic review of prevalence and risk factors

**DOI:** 10.3389/fpubh.2025.1711097

**Published:** 2025-11-24

**Authors:** Wei Zhou, Kaizhi Gu, Yiyu Chen

**Affiliations:** 1 Department of Art, Southeast University, Nanjing, Jiangsu, China; 2 Department of Art, Nanjing University of the Arts, Nanjing, Jiangsu, China

**Keywords:** musculoskeletal disorders, older hospital cleaners, prevalence, risk factors, systematic review

## Abstract

**Introduction:**

Hospital cleaning involves unique risk factors including chemical exposures, infection control procedures, and physically demanding tasks that may have greater impact on older workers compared to their younger counterparts. While existing systematic reviews have examined musculoskeletal disorders among cleaning workers, none have specifically focused on older hospital cleaners. With global workforce aging and increasing employment of older adults in healthcare settings, understanding musculoskeletal disorder prevalence and specific risk factors in this population is essential for developing targeted prevention strategies. The primary objective of this systematic review is to determine the prevalence of musculoskeletal disorders among older hospital cleaners and identify associated risk factors.

**Methods:**

We searched PubMed, MEDLINE, Web of Science, and Google Scholar from inception to July 11, 2025. Two reviewers independently screened titles, abstracts, and full texts. Data extraction included study characteristics, participant demographics, prevalence data, and risk factors. Quality assessment was conducted using the Joanna Briggs Institute checklist.

**Results:**

Eleven studies were included. Among older hospital cleaners (aged 50+ years), musculoskeletal disorder prevalence ranged from 49.1% to 68.96% across age-stratified studies. Several factors appeared to be associated with MSD risk in this population, including work demands, individual factors (age, gender, inadequate rest time, stress, and posture), technology-related factors, and organizational factors.

**Discussion:**

This systematic review identified multiple risk factors for musculoskeletal disorders among older hospital cleaners. The findings provide evidence to inform occupational health policies and targeted prevention strategies for this vulnerable population.

## 1 Introduction

Musculoskeletal disorders (MSDs) encompass a range of inflammatory and degenerative conditions affecting muscles, tendons, joints, ligaments, and other musculoskeletal structures (1). As one of the most commonly reported occupational injuries globally (2–4), MSDs are characterized by high prevalence (5). These conditions are associated with absenteeism and early retirement, which negatively affect daily activities, productivity, and quality of life, and in severe cases may lead to disability (6–8). Beyond the direct health impacts, MSDs impose a substantial economic burden: indirect costs often exceed direct medical expenses, making MSDs among the most expensive occupational diseases worldwide (9–11). When combined with productivity losses and increased strain on public healthcare systems (12–16), MSDs have become an urgent global public-health challenge (17).

Previous studies have identified four major categories of risk factors for work-related musculoskeletal disorders: biomechanical, individual, mental, and organizational (2, 5). Biomechanical risk factors include poor working postures (18–20), repetitive motions (21, 22), vibrations (23), heavy physical demands such as lifting or carrying loads (18, 20, 24, 25). Individual risk factors include age (26), gender (27), smoking (18), BMI (18, 24, 28, 29), sleep habits (5, 30), and prior medical history (29). Mental risk factors include negative emotions (31) and workload (18, 32, 33). Organizational risk factors include shift work (5, 34) and lack of workplace support (22, 25). Collectively, these determinants form a multidimensional framework encompassing biomechanical, individual, mental, and organizational domains. These four categories of risk factors are particularly pronounced among hospital cleaners, who face unique occupational challenges due to the nature of their work environment and task demands.

The vulnerability of hospital cleaners to MSDs has been further amplified by demographic changes in the workforce (35). In recent years, global population aging has prompted retirement-policy reforms across various regions (36). These policies have extended retirement age (37), particularly for individuals in physically demanding occupations. As a result, the proportion of older workers in the service industry has steadily increased (38, 39). Hospital cleaning work have become an increasingly common employment option among older adults (40). According to the U.S. Bureau of Labor Statistics, hospital cleaning positions in general medical and surgical hospitals constitute one of the largest employment sectors (41). The proportion of cleaners working in hospitals and educational institutions is significantly higher than that of other types of cleaners (42). In this review, the term “hospital cleaners” refers specifically to cleaning workers in general medical and surgical hospitals.

Given their increasing numbers and combined exposure to multiple risk domains, MSDs pose a critical occupational-health challenge for hospital cleaners (6, 43, 44). Healthcare cleaning personnel bear substantial responsibility for disrupting infection-transmission chains (6, 36, 45–47). Consequently, they must perform cleaning tasks at higher frequencies, with high-risk hospital areas sometimes requiring cleaning every 4 h (48). Moreover, the 24-h operation demands of hospitals necessitates prolonged and irregular shifts, further elevating musculoskeletal risk compared with cleaners in other environments (44). In routine operations, their standard duties include preparing cleaning products and sanitizing various surfaces throughout the hospital (44, 49). These tasks involve high physical demands, including prolonged standing, repetitive movements, distance walking, and sustained cardio-respiratory exertion (50). This growing demographic shift underscores an urgent need to understand the prevalence and risk factors of MSDs within this essential yet understudied workforce.

Multiple studies have demonstrated a high prevalence of MSDs among hospital cleaners, particularly affecting the back, shoulders, neck, and knees (1, 5, 22, 26, 27, 51, 52). Within the cleaning workforce, there is a substantial proportion of older individuals and those with limited skills (40, 53–55). In particular, individuals aged 50–59 years face higher risks of developing MSDs (56). Numerous studies have shown that risk factors such as age, gender, poor posture, and prior medical history are associated with musculoskeletal disorders among older hospital cleaners (26, 55). Six systematic reviews on MSDs among cleaners have been published between 1979 and 2024, consistently identifying cleaning workers as a high-risk occupational group for MSDs. The focus of these reviews has evolved from identifying MSD risk factors (57, 58) to developing intervention strategies (53, 59–61). However, despite the rapidly aging workforce in healthcare cleaning services, the intersection between age-related vulnerability and hospital-specific exposures remains poorly understood, representing a critical research gap.

There is currently no universal consensus regarding the definition of “older.” Public policies apply varying age thresholds ranging from 45 to 65 years (62, 63). For instance, the World Health Organization defines “older adults” as 65 and above in general-population contexts (64). Such thresholds are commonly tied to pension eligibility in different national contexts (65). However, aging is a dynamic, multidimensional process (66). In occupational contexts, the concept of “older workers” extends beyond chronological age to include organizational and career-stage dimensions (67). Its social definition continues to evolve in different workplace contexts (65). Empirical evidence showed that decision-makers perceive workers as “older” at a mean age of 52.40 years, with younger decision-makers (≤35 years) setting this threshold even lower at 50.91 years (65).

Given this complexity, this review establishes 50 years as the age criterion based on three lines of evidence from occupational musculoskeletal health research. First, the Global Burden of Disease study reported a significant increase in MSDs case numbers and prevalence specifically among the 50–59 age group (56). This indicates that workers aged 50+ who remain employed represent a critical population for understanding how to support continued productive engagement despite age-related health challenges. Second, the prevalence and risk factors of MSDs across different body regions among older hospital cleaners may differ substantially from those observed in younger workers (68). Third, this threshold aligns with age-stratifications practices commonly used in occupational-health studies (69–71). Despite the growing body of literature on MSDs among hospital cleaners, to our knowledge, no systematic review has specifically synthesized evidence on prevalence and risk factors among older workers (≥50 years) in this occupation. This is a critical gap given the unique vulnerabilities of aging workers and the increasing proportion of older adults in the cleaning workforce.

Therefore, this systematic review aims to (1) synthesize the prevalence of musculoskeletal disorders among older hospital cleaners, (2) identify associated risk factors, and (3) provide recommendations for intervention strategies in general medical and surgical hospitals.

## 2 Methods

This systematic review was conducted following the Preferred Reporting Items for Systematic Reviews and Meta-Analyses (PRISMA) statement (72). The PRISMA checklist is listed in Supplementary material S2.

The study protocol was prospectively registered in the *International Prospective Register of Systematic Reviews* (PROSPERO; registration number: CRD420251102052). Initially, the age threshold for inclusion was set at 55 years; however, due to the limited number of relevant articles, the threshold was adjusted to 50 years to ensure adequate study inclusion. Studies involving hospital cleaners aged 50 years and above were included in this review.

### 2.1 Data sources

A comprehensive literature search was performed in PubMed, MEDLINE, Web of Science, and Google Scholar from inception to July 11, 2025.

### 2.2 Inclusion and exclusion criteria

We applied the PICO framework to establish inclusion criteria (73): Population (P): Hospital cleaning workers aged 50 years and older employed in general medical and surgical hospitals. Studies providing age-stratified data for hospital cleaning workers aged 50+ within mixed-age populations were also included; Intervention (I): Hospital cleaning work activities; Comparison (C): Any comparison group or no comparison; Outcomes (O): Musculoskeletal disorder prevalence and risk factors associated with musculoskeletal disorders.

During the screening process, studies were excluded according to the following criteria. Records not relevant to the topic were removed during initial database searches, and duplicate records across databases were eliminated. Studies without accessible full text were excluded. To ensure inclusion of primary research only, all review or synthesis papers were excluded. Non-English publications were excluded from the analysis. Finally, studies with participants below 50 years of age or reporting mean ages under 50 years without age-stratified data were excluded to maintain focus on older hospital cleaners.

### 2.3 Search strategy

A systematic search was conducted in PubMed, MEDLINE, Web of Science, and Google Scholar from inception to July 11, 2025. Search strategies were tailored for each database and are presented in Supplementary Table S1.

### 2.4 Literature screening and data extraction

Two reviewers independently screened all titles, abstracts, and full-text articles using inclusion and exclusion criteria. Disagreements between reviewers were resolved through discussion with a third reviewer to reach consensus. Additionally, we used a study characteristics form to capture prevalence data for musculoskeletal disorders and to identify risk factors.

### 2.5 Data items

Two reviewers extracted data including country, study design, study period, hospital setting, sample size (% female), primary outcome, MSD assessment method; risk factors, prevalence (%), sample selection, confounding control, and limitations.

### 2.6 Risk of bias assessment

The methodological quality of each included study was assessed using the Joanna Briggs Institute (JBI) critical appraisal checklist for cross-sectional studies (74). This checklist has been widely used for assessing the quality of studies included in systematic reviews (58, 75). Each item was scored as “Yes,” “No,” or “Unclear.” Two reviewers independently conducted the assessments, and consensus was reached through discussion when disagreements occurred. The JBI quantitative critical appraisal tool was applied to assess the risk of bias in the included studies (76). Included studies were classified based on the number of JBI checklist items answered with “Yes”: studies answering 0–3 items were categorized as “poor quality,” those answering 4–6 items as “moderate quality,” and studies answering 7–8 items were considered “high quality” (58).

### 2.7 Task categorization

Given the heterogeneity in task descriptions, two reviewers independently extracted all reported cleaning tasks from the included studies. Through iterative discussion and consensus building, we grouped similar tasks according to three criteria: (1) primary biomechanical demands, (2) equipment and tools used, and (3) work environment characteristics. Any disagreements between reviewers were resolved through discussion with a third reviewer until consensus was achieved.

### 2.8 Data synthesis

Owing to substantial heterogeneity among the included studies, meta-analysis was not feasible, and a descriptive synthesis approach was employed instead. This heterogeneity was primarily attributed to several factors: (1) the wide age range of participants aged 50 years and above across studies; (2) only six studies provided age-stratified data, with two lacking prevalence data; and (3) among the remaining four studies with age stratification and prevalence data, two assessed musculoskeletal disorders over the last 7 days, while two examined prevalence over the last 12 months, making statistical pooling inappropriate. Data extraction focused on study design, participant demographics, outcome measures, prevalence rates, and identified risk factors. Results were presented using narrative synthesis.

During data synthesis, a conceptual model grounded in the Person–Environment (P–E) Fit theory was constructed to illustrate the interactions between work demands, individual characteristics, and environmental factors contributing to musculoskeletal disorders among older hospital cleaners (Figure 2). This conceptual framework was developed inductively based on the synthesis of identified risk factors and existing theoretical foundations from occupational health and organizational psychology literature.

## 3 Results

### 3.1 Literature search results

**Figure 1:**
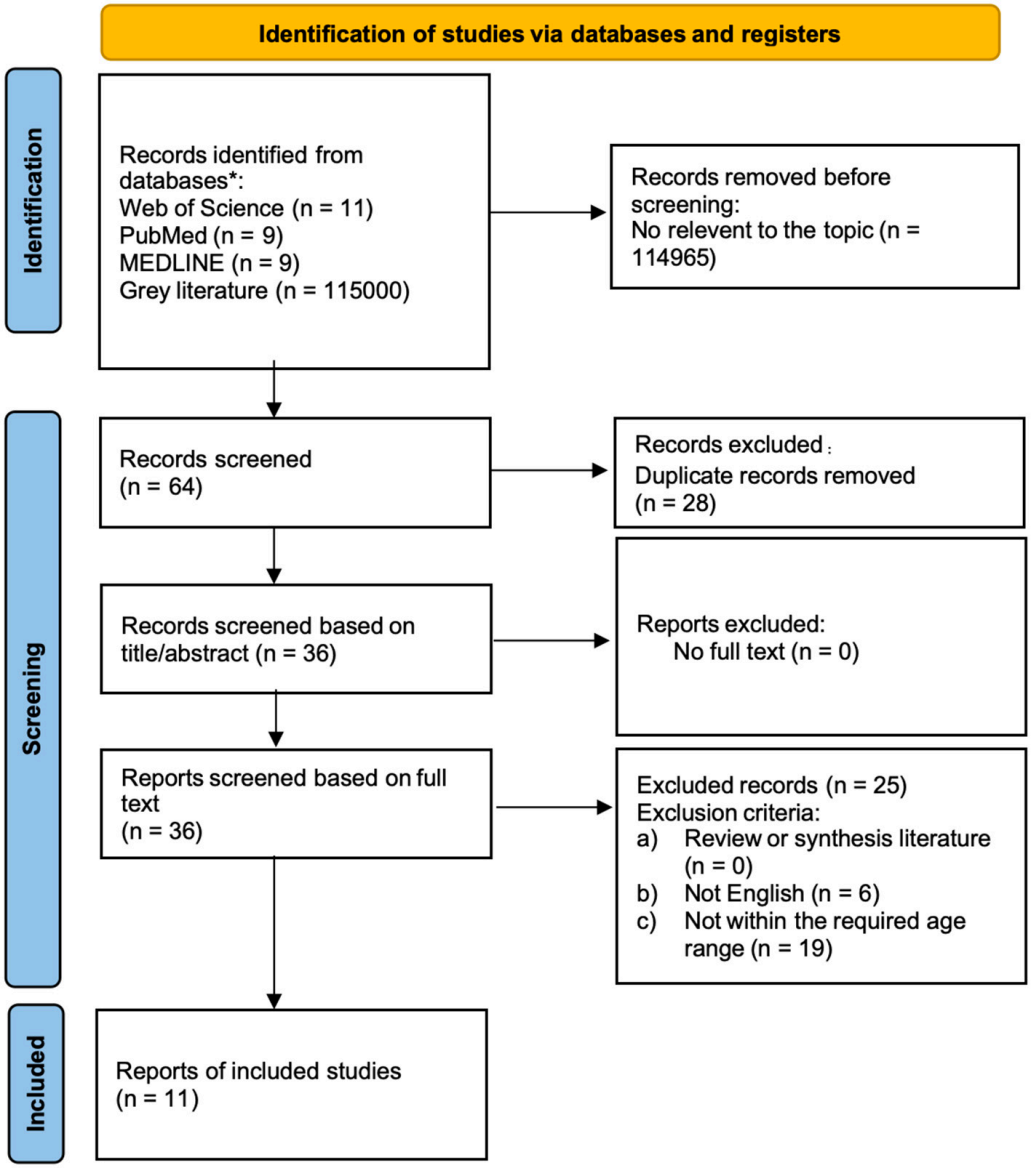
PRISMA flow diagram (72).

We searched multiple databases including PubMed, MEDLINE, Web of Science, and Google Scholar from inception to July 11, 2025. After screening and eligibility assessment, 11 studies met the inclusion criteria and were included in this review (Figure 1).

### 3.2 Baseline characteristics

Table 1 presents the baseline characteristics of the included studies, including country, study design, study period, hospital setting, sample size, age, sample size, proportion of females (%), primary outcome, MSD assessment method, risk factors, prevalence (%), sample selection, confounding control, and study limitations.

**Table 1:** Characteristics of included studies.

**Study (First author, Year)**	**Country**	**Identified risk factors**	**Prevalence of MSD (%)**	**Action level**
Prevalence and factors associated with musculoskeletal pain among hospital cleaning staff. (6)	Brazil	• Staffing dissatisfaction • Alcohol use • Self-medication • Sedentary lifestyle • Smoking • Medication use • Self-medication • Married status • Alcohol use • Sedentary lifestyle • Sleep < 8 h/day	• Lower back: 42.3% • Ankle/Foot: 28.2% • Wrist/Hand: 27.5% • Upper back: 25.5% • Shoulder: 23.5%	**High action level** The study revealed widespread MSD symptoms and statistically significant associations between multiple modifiable lifestyle and organizational factors. Authors explicitly recommended implementing health education programs and ergonomic process improvements to minimize exposure to musculoskeletal pain.
Physical ergonomic assessment in cleaning hospital Operating rooms based on inertial measurement units. (77)	France	• Manual handling of heavy objects such as operating tables, stretchers, and carts. • Prolonged trunk flexion and overhead reaching during cleaning tasks. • Awkward arm postures and repetitive upper-limb movements.	• Neck (66.7%) • Shoulders (66.7%) • Elbows (11.1%) • Wrists (44.4%) • Upper back (44.4%) • Lower back (77.8%)	**High action level** IMU-based ergonomic assessment revealed very high biomechanical exposure, particularly for the trunk and upper limbs, corresponding to REBA/RULA high-risk zones. The authors emphasized the need for redesigning cleaning workflows (e.g., mechanical lifting aids, reduced trunk flexion angles, and alternating work postures) to lower musculoskeletal load.
Women at work despite ill health: diagnoses and pain before and after personnel support. A prospective study of hospital cleaners/home-help personnel with comparison groups. (83)	Sweden	• Persistent pain despite working.	• Shoulder girdle: 51.1% • Low back: 22.2% • Hip muscles: 22.2% • Rotator cuff: 17.8% • Neck muscles: 15.6%	**High action level** This study implemented a 12-month personnel support intervention. After intervention, 73.9% of hospital cleaners showed improvement, compared to only 27.3% in the control group, indicating the program's effectiveness. The authors recommend sustained workplace ergonomic and psychosocial support to prevent chronic MSD progression and long-term sick leave.
Prevalence and factors associated with musculoskeletal pain in hospital cleaning workers. (84)	Brazil	• Age 19–34 years • Lack of leisure time.	Overall MSP: 70.1% (*n* = 110 of 157). • Strong/unbearable pain: 25.5%.	**High action level** Although no direct occupational factors were statistically significant, the high prevalence of severe pain among young workers and those lacking leisure suggests an urgent need for ergonomic redesign, workload reduction, and health promotion initiatives.
The prevalence and risk factors of musculoskeletal disorders among subcontracted hospital cleaners in Thailand. Journal of Health Research. (44)	Thailand	• Male gender • Severe stress • History of injuries • Mopping posture • Task duration for 2–4 and for more than 4 h	7-day MSDs: 73.9% 12-month MSDs: 81.9% • Lower back: 57.7% • Shoulder: 52.6%	**Very high action level** The study identified severe biomechanical and psychosocial burdens among subcontracted hospital cleaners, particularly related to prolonged forward bending, excessive working hours.
Factores asociados a trastornos musculoesqueléticos en trabajadores de limpieza del servicio de emergencia de un hospital terciario. (78)	Peru	No factors associated with musculoskeletal disorders were found	Overall MSDs: 93.02% • Lumbar pain: 65.12% • Dorsal (back) pain: 47.29% • Neck pain: 37.21% • Elbow/forearm pain: 13.18%	**Moderate-to-high action level** Although no specific predictors reached statistical significance in the adjusted model, the very high prevalence of multi-site pain (93.02%) indicates a critical ergonomic concern.
The association of socioeconomic status and psychosocial and physical workplace factors with musculoskeletal injury in hospital workers. (80)	USA	• Occupation group (other clinical workers) • Effort-reward imbalance • Ergonomic exposures (lower body strain)	Not directly reported as a percentage but described as high incidence among hospital workers with significant pain.	**High action level** Effort-Reward Imbalance (ERI) identified as a significant psychosocial risk factor for MSDs. Organizational and ergonomic interventions required to reduce psychosocial and physical load.
Janitor ergonomics and injuries in the safe workload ergonomic exposure project (SWEEP) study. (81)	USA	• Heavy ergonomic workload • High task frequency	N/A	**High action level** Heavy ergonomic workload positively associated with injury occurrence. Intervention needed to reduce task frequency/intensity and improve equipment design and work organization.
Ergonomic analysis of workplace furniture in hospitals: a public hospital example. (82)	Turkey	• Workplace furniture ergonomic factors • Environmental conditions	N/A	**Medium action level** Participants showed partial satisfaction with ergonomic conditions. Environmental and furniture design issues such as inadequate lighting, limited workspace, and poor thermal comfort reduced comfort and efficiency, indicating the need for ergonomic redesign and training.
Musculoskeletal ill health amongst cleaners and recommendations for work organizational change. (42)	UK	• Manual handling • Awkward postures • Equipment factors • Work organization factors	74 % reported musculoskeletal pain or discomfort in the past year. • Lower back: 46 % • Neck: 33 % • Knees: 24 % • Shoulder: 23 % • Wrist/hand: 22 %	**High action level** Organizational improvements such as teamwork, job rotation, and better training were recommended to reduce these risks and promote musculoskeletal health.
Nonfatal occupational injury rates and musculoskeletal symptoms among housekeeping employees of a hospital in Texas. (79)	USA	• Slip/trip/fall • Material handling	64.2% overall • Lower back: 49% • Wrists: 43% right, 35% left • Knees:34%−35% • Shoulders: 25% • Neck:16%	**High action level** The authors recommended ergonomic redesigns, anti-slip measures, relaxation training, and participatory organizational interventions to mitigate these risks.

Among the 11 included studies, seven (63.6%) were conducted in Global North regions, comprising three studies from the United States and one each from France, Sweden, the United Kingdom, and Turkey. The remaining four studies (36.4%) were conducted in Global South regions, including two studies from Brazil and one each from Thailand and Peru (Table 1).

The majority of studies (*n* = 8, 72.7%) were conducted in public healthcare settings. Five studies were conducted in public hospitals, two in public university hospitals, and one in the broader public healthcare sector; hospital setting information was unavailable for three studies.

Study designs included eight cross-sectional studies (72.7%), two prospective cohort studies (18.2%), and one case-control study (9.1%).

Publication years ranged from 2001 to 2024, spanning two decades of research. The distribution showed three studies (27.3%) from 2001–2010, while the 2011–2020 and 2021–2024 periods each contributed four studies (36.4%), indicating sustained and increasing research interest in recent years.

Age distributions varied widely across studies, though specific sample sizes for older worker subgroups were not consistently reported across all studies. Gender distribution was available for nine studies, revealing a predominantly female workforce with female participation rates ranging from 12.5% to 100%.

The primary outcomes of the included studies were grouped into two main categories. Eight studies (72.7%) focused on determining the prevalence of musculoskeletal disorders or pain and assessing multidimensional risk factors, including economic, organizational, psychosocial, and physical determinants among hospital cleaning staff. The remaining three studies (27.3%) examined ergonomic assessment.

### 3.3 Risk of bias

We used the JBI checklist to evaluate the risk of bias in included studies (Table 2). The risk of bias assessment comprised eight questions (58). Two reviewers independently assessed each study, with disagreements resolved through discussion. Detailed risks of bias assessments for individual studies are summarized in Table 3. The assessment revealed that all 11 included studies demonstrated high and moderate quality. The main source of bias was related to confounding factor management. Six studies failed to clearly identify potential confounders or implement appropriate statistical adjustment strategies.

**Table 2:** The JBI checklist to evaluate the risk of bias of included studies.

**No**.	**Included study**	**Q1**	**Q2**	**Q3**	**Q4**	**Q5**	**Q6**	**Q7**	**Q8**	**Total**	**Quality**
1	(68)	Y	Y	Y	Y	Y	N	Y	Y	7/8 (87.5%)	High
2	(77)	Y	Y	Y	Y	U	N	Y	Y	6/8 (75%)	Moderate
3	(83)	U	Y	Y	Y	Y	U	Y	Y	6/8 (75%)	Moderate
4	(84)	Y	Y	Y	Y	Y	Y	Y	Y	8/8 (100%)	High
5	(44)	Y	Y	Y	Y	Y	Y	Y	Y	8/8 (100%)	High
6	(78)	Y	Y	Y	Y	Y	Y	Y	N	7/8 (87.5%)	High
7	(80)	Y	Y	Y	Y	Y	Y	Y	Y	8/8 (100%)	High
8	(81)	Y	Y	Y	Y	Y	Y	Y	Y	8/8 (100%)	High
9	(82)	Y	Y	Y	Y	U	N	Y	Y	6/8 (75%)	Moderate
10	(42)	Y	Y	Y	Y	U	N	Y	Y	6/8 (75%)	Moderate
11	(79)	U	Y	Y	Y	Y	U	Y	U	5/8 (62.5%)	Moderate

Y, Yes; N, No; U, Unclear.

**Table 3:** The prevalence of MSDs among older hospital cleaners.

**Study**	**Age group**	**Period**	**Prevalence**
(68)	49–62 years	The last 7days	Neck (13.5%); shoulders (17.3%); elbows (25%); wrists/hands (23.1%); upper back (42.3%); lower back (42.3%); hip/thighs (30.8%); knees (34.6%); ankles/feet (21.2%)
(79)	39–58 years; >59 years	The last 12 months	68.96%; 66.66%
(84)	45–60 years	The last 7days	57.1%
(44)	40–63 years	The last 12 months	49.1%

### 3.4 Outcome measurement

Five studies employed the Nordic Musculoskeletal Questionnaire (NMQ) or the Standardized Nordic Questionnaire (SNQ) to assess MSD prevalence and related factors (44, 68, 77–79). Both NMQ and SNQ are widely used to collect data on work-related musculoskeletal problems or pain in specific body regions. Four studies utilized self-reported structured questionnaires to conduct ergonomic assessments or workplace evaluations (42, 80–82). Additionally, two studies employed the Visual Analogue Scale (VAS) to measure individuals' perceived intensity or changes in specific sensations, symptoms, or conditions (83, 84).

Due to substantial heterogeneity among study participants, a meta-analysis was not feasible; therefore, a descriptive analysis was employed to synthesize the results. All 11 articles included hospital cleaners aged 50 years and above. Among these, six studies provided age-stratified data (44, 68, 79, 81, 82, 84), of which four studies reported prevalence rates (Table 3). Results from the four studies with age-stratified data showed that older hospital cleaners (aged 39–62 years across studies) had overall MSD prevalence rates ranging from 49.1% to 68.96%. Prevalence rates varied across different body regions, with the upper back and lower back showing the highest prevalence rates, both reaching 42.3%. Importantly, different studies employed varying time windows (7 days vs. 12 months), which may affect prevalence comparisons. The 12-month prevalence was generally higher than the 7-day prevalence, which is consistent with the chronic and recurrent nature of MSDs in this population.

## 4 Discussion

Previous systematic reviews on musculoskeletal disorders among cleaners have established theoretical foundations. One review developed a conceptual model that identified potential factors contributing to musculoskeletal disorders among cleaners (53). This model posits that load, tissue response, and modifying factors jointly govern musculoskeletal adaptation and injury risk, shaping cleaners' health and work ability (53). Subsequently, another review built upon this foundation to identify five key components of work structure (individual, technology, organization, environment, and outcome) based on balance theory (61).

However, no systematic reviews have specifically focused on MSDs among older hospital cleaners. Additionally, previous models have not adequately addressed the dynamic relationship between cleaner characteristics and MSDs. Older workers generally face age-related degenerative changes, with declining muscular strength being a key manifestation of this deterioration. Previous literature has demonstrated that muscular strength may be a critical factor in determining MSD risk when performing physically demanding work tasks. Lower muscle strength in vulnerable body regions may increase the risk of MSDs during heavy physical labor (85). Conversely, higher muscle strength may enhance cleaners' tolerance for heavy physical work and provide a protective balance against the development of musculoskeletal symptoms (86).

**Figure 2:**
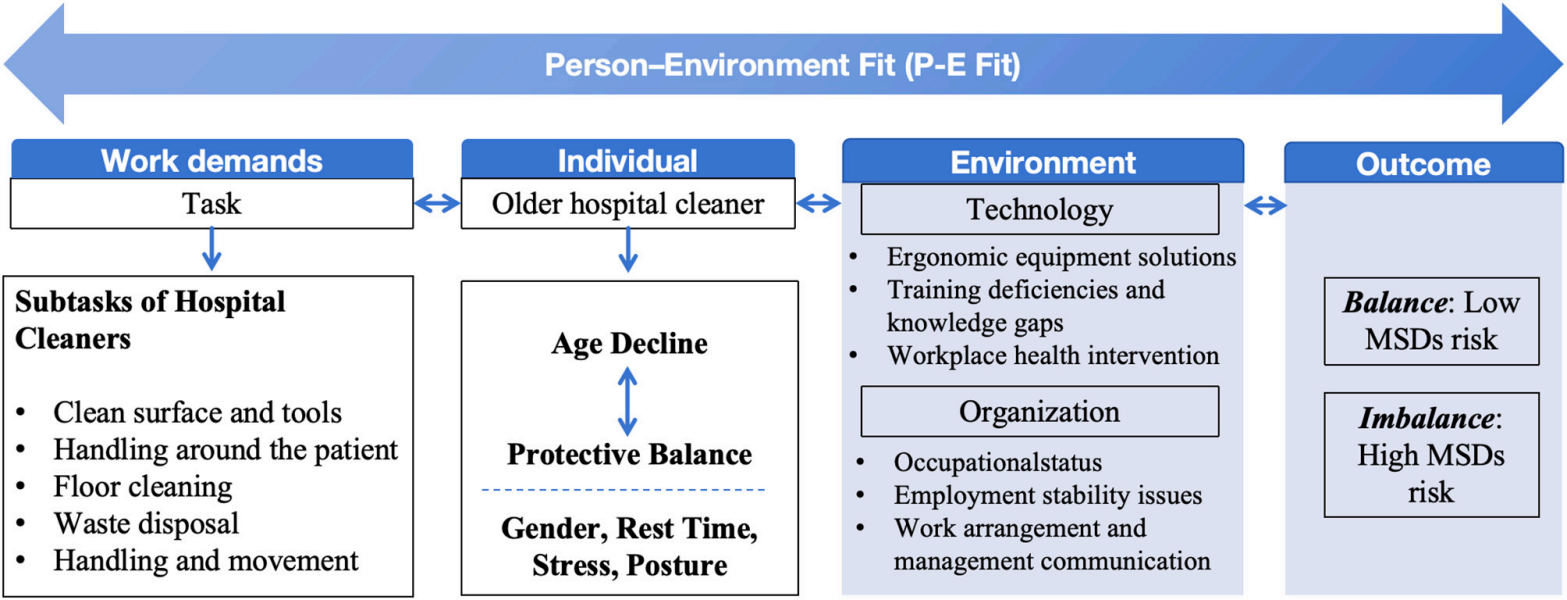
A conceptual model of musculoskeletal disorder risk factors in older hospital cleaners based on Person-Environment Fit (P-E Fit) theory.

This suggests that an individual's adaptive capacity when performing work tasks may influence MSD outcomes. Based on these findings, this study developed a conceptual model based on Person-Environment Fit (P-E Fit) theory (Figure 2). P-E Fit is typically defined as the compatibility between individual and work environment characteristics (87). The P-E Fit model is widely applied in organizational psychology and occupational health fields, and has increasingly been used in work-health relationship research. Multiple studies have emphasized that good P-E Fit can help workers adapt to work and return to work through dynamic, phased, and cyclically adjustable processes, ultimately reducing the prevalence of MSDs (88–90).

The population aged 50 and above generally corresponds to individuals from the later Baby Boomer and early Generation X cohorts. A study based on the P-E Fit model explored person-environment fit from three perspectives: individual-work, individual-group, and individual-supervisor relationships (91). This study indicated that person-environment fit for individuals over 50 can be predicted through person-job fit (91), emphasizing that ensuring alignment between job content and characteristics of this population is critical (92).

### 4.1 Subtasks of hospital cleaners

Hospitals encompass diverse workspaces and represent high-risk environments for musculoskeletal disorders (80). Each phase of hospital cleaning work presents specific MSD risk factors (93). One study identified eight daily tasks performed by cleaners, including emptying large trash bins, emptying small (less than 25 pounds) trash bins, mopping, vacuuming, dusting, cleaning toilets, cleaning mirrors, and cleaning sinks; Through Rapid Entire Body Assessment scoring, all tasks were classified as high-risk categories (81). The study by Bello et al. (49) showed that hospital cleaning tasks included preparing cleaning solutions, floor cleaning, window cleaning, mirror cleaning, toilet cleaning, sink cleaning, and countertop cleaning. A study conducted by Laithaisong et al. (44) categorized cleaning work into five main space subcategories: inpatient department, outpatient department, high-level space, office/dormitory, and waste disposal. Another study listed subtasks including waste disposal, lighting management, handling situations around patients, cable and pipe management, patient transport, surface and tool cleaning, various object movement, floor cleaning, box lifting, water tank handling, high-pressure cleaning, operating table movement, stretcher movement, operating table cleaning, operating table disassembly, sheet movement, and cart movement (77).

**Figure 3:**
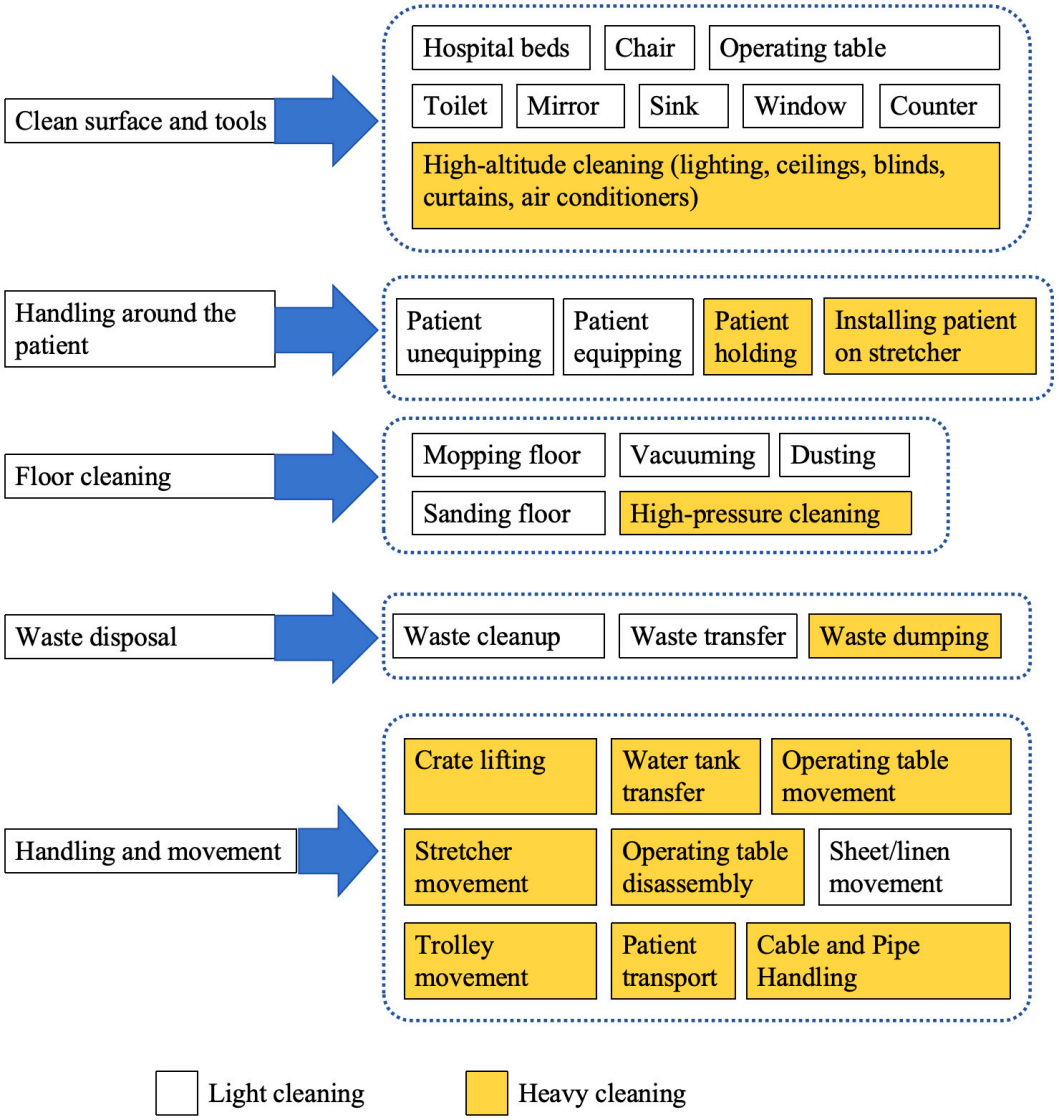
Five main categories of hospital cleaning tasks.

Given the lack of consensus regarding hospital cleaners' work tasks, we synthesized findings from multiple studies to identify five main categories of hospital cleaning tasks: surface and tool cleaning, handling around patients, floor cleaning, waste disposal, and handling and movement (Figure 3).

Each cleaning task has its unique musculoskeletal disorder risk factors. For example, dusting and scrubbing tasks often involve awkward postures such as overhead reaching, bending, kneeling, squatting, and wrist flexion (93). Based on physical demands, cleaning tasks are typically categorized as light or heavy cleaning in recent occupational studies. Light cleaning includes dusting and cleaning countertops, while heavy cleaning encompasses tasks with higher physical demands (94). One study suggested that high physical demand activities are the primary risk sources for musculoskeletal disorders among hospital cleaners (84). Therefore, heavy cleaning tasks require more attention and intervention measures, including equipment improvements and training in relevant ergonomic knowledge.

### 4.2 Individual factors associated with MSDs

#### 4.2.1 Age

Previous studies have demonstrated that advancing age is associated with degeneration of the musculoskeletal system and is one of the most common risk factors for MSDs (64, 95). However, regarding hospital cleaners, the impact of age on musculoskeletal disorders has yielded mixed conclusions.

Some studies support age as a risk factor for MSDs. Older cleaners face a contradiction between high physical work demands and declining physical work capacity, making them more susceptible to musculoskeletal injury risks (96). Age-stratified analysis showed that the prevalence of MSDs in the 39–62 age group ranged from 49.1% to 68.96%, while hospital cleaners over 59 years old had a prevalence rate of 66.66%, with particular attention needed for upper back and lower back MSD risks (42, 68, 79). Another study showed that healthcare workers aged 47 and above had approximately twice the risk of developing upper extremity musculoskeletal pain compared to younger employees aged 34 and below (97).

However, other studies have reached opposite conclusions, suggesting that age may sometimes act as a protective factor, which may be explained by lighter work tasks and greater work experience among older cleaners. One study showed that the MSP prevalence in the 45–60 age group (57.1%) was lower than in the 19–34 age group (76.5%) and 35–44 age group (78.0%), which may be because younger employees often handle more physically demanding activities, while older cleaners possess more extensive professional experience (84). This finding is consistent with a US study showing that hospital cleaners aged 39–58 years (68.96%) had higher prevalence rates than those over 59 years (66.66%), while the 18–38 age group had lower prevalence (51.85%) (79). This pattern may be explained by the latency period from musculoskeletal pain symptoms to diagnosis, which can span months to years (78). One study identifie the middle-age period (35–50 years) as a critical prevention window for musculoskeletal disorders, when some physical problems begin to manifest but workers have not yet left their positions (98). Some hospital cleaners choose to continue working despite ill health (83).

Organizations and governments could consider providing occupational screening for hospital cleaners around age 35 and implement preventive interventions during this period. Such screening and interventions may help avoid long-term sick leave in the workplace. During earlier career stages, musculoskeletal training and guidance should be incorporated to promote proper body posture (84).

Some studies found no significant association between age and MSDs (44, 78). One possible explanation is that the smaller number of older cleaners reduced statistical power. Additionally, the selection of adjustment variables may have been overly conservative, which could partially explain why significant risk factors were not identified. Another possibility is the healthy worker effect, where high-risk individuals may have already left employment.

Age-related physiological decline may further amplify the risk of WMSDs among older cleaners. However, the findings across studies remain contradictory (79). Several investigations suggest that WMSD prevalence increases with age, peaks during midlife and then declines thereafter (68, 84). This apparent paradox can be partly explained by the “healthy worker effect,” whereby workers who cannot tolerate pain or physical strain leave employment earlier, resulting in a relatively healthier remaining workforce (99). In addition, older cleaners who remain employed may benefit from lighter work assignments, accumulated experience, and adaptive coping strategies that mitigate physical strain (84). Considering the latency between symptom onset and clinical diagnosis, as well as older workers' higher pain tolerance and greater economic dependence on work, a considerable number of symptomatic but untreated individuals may exist in this group (78, 83, 98). Therefore, older hospital cleaners represent a distinct subgroup shaped by both health selection and age-related physiological decline, whose WMSD patterns and risk factors differ from those of younger workers. Future epidemiological studies focusing specifically on this population are needed to better understand their true disease burden and inform targeted preventive measures.

#### 4.2.2 Gender

Evidence on gender-related MSD risk remains inconsistent across studies (78). Higher MSD prevalence among females may be attributed to gendered division of labor, lower upper-body strength, and additional domestic workload, which increase cumulative physical strain (84). Conversely, male cleaners may experience greater exposure to heavy lifting and forceful exertion, leading to different injury patterns (44). These gendered risk patterns align with findings from other physically demanding occupations, such as surgeons (100), dentists (101) and other occupation (102, 103), where gender-specific task allocation and ergonomic mismatch contribute to differential MSD risks (104).

A study further showed that the two most common types of injuries or disorders among both male and female were sprains/strains and general soreness or pain. Specifically, male cleaners had higher rates of sprains/strains or tears; female cleaners more frequently experienced bruises and contusions (105). These distinctions likely reflect the division between heavy and light cleaning duties.

Future preventive strategies should incorporate gender-sensitive approaches. For female cleaners, interventions should focus on mitigating cumulative musculoskeletal strain and preventing contusions, particularly among those balancing work and childcare responsibilities; for male cleaners, emphasis should be placed on safe lifting techniques and reducing acute sprain and strain injuries.

#### 4.2.3 Rest time

One study indicated that insufficient rest time was associated with a higher prevalence of musculoskeletal disorders. After multivariable adjustment: no leisure time, PRa = 1.14 (95% CI = 1.03–1.27, *p* = 0.013), occasional leisure time, PRa = 1.10 (95% CI = 1.02–1.20, *p* = 0.021) (84). These findings suggest a dose-response relationship between rest time and MSD risk, highlighting that insufficient recovery may lead to the accumulation of muscle fatigue and delayed tissue repair. This is consistent with evidence from other physically demanding occupations (e.g., nursing and manufacturing), which has shown that inadequate rest disrupts recovery cycles and increases the likelihood of chronic musculoskeletal pain (106–110).

#### 4.2.4 Stress

In physically demanding work, stress can lead to fatigue while increasing the biomechanical load on muscles and tendons (44). One study indicated that hospital cleaners with a history of severe stress and injury in the past year had higher associations with MSDs occurrence (44), suggesting that screening rates should be increased for this population.

Additionally, the hospital work environment presents unique psychosocial factors, including pressures from the fast-paced medical environment and life-and-death work responsibilities (80). These findings are consistent with other occupational studies showing that high job stress, shift work, and insufficient social support can exacerbate musculoskeletal symptoms through chronic physiological stress responses (5, 111–114).

#### 4.2.5 Posture

A Thai study identified repetitive forward-bending postures of the back as one of the risk factors for MSDs (44). Research has shown that high physical load, repetitive movements, and uncomfortable postures increase the risk of developing musculoskeletal symptoms (51). Specifically, a French study used Rapid Upper Limb Assessment (RULA) to evaluate the ergonomic scores of cleaners' bodies, finding that elbows and forearms face higher risk levels in hospital cleaning work. Tasks such as “moving stretchers” and “moving operating tables” involve heavy object displacement, exposing cleaners to more adverse postures (77). However, some mechanical assistive devices can help alleviate these problems (115). Poor posture leads to uneven load distribution and localized muscle fatigue, explaining its strong link with MSDs. To address this issue, improvements in mop handle design and training for cleaning staff on proper work postures and tool usage are necessary. Similar ergonomic interventions in healthcare and industrial settings have demonstrated reductions in MSD prevalence (116, 117), supporting the need for posture-specific training among hospital cleaners.

### 4.3 Technology factors associated with MSDs

New technologies and ergonomic equipment can mitigate individual risks in the cleaning occupation (93). For example, a US study designed an improved hospital waste bin that limited the degree of shoulder and trunk flexion required to remove garbage bags, thereby reducing repetitive overhead movements and thereby decreasing the risk of muscle strains or sprains (118). Additionally, another study reported ergonomic solutions for long-handled tools, gloves, vacuum cleaners, mops, buckets, furniture, and waste bins. These improved tools can help cleaners minimize awkward postures and repetitive strain (93). These findings suggest that healthcare facilities should prioritize ergonomic equipment procurement and regular technology upgrades to create safer working environments for cleaning staff.

Training and supervision are fundamental safeguards for ensuring hospital cleaners' health. A Turkish cross-sectional study revealed that most participants in public hospitals had only partial understanding (52.6%) or complete lack of understanding (29.9%) of ergonomic working conditions, with over half (56.2%) expressing a desire to receive relevant training (82). Similarly, a UK study found that approximately 26% of workplace cleaners indicated inadequate cleaning training (42). Additionally, a study targeting female cleaning workers demonstrated that workplace health intervention programs (ergonomic equipment, professional instruction, and designated work time) were more cost-effective than home exercise guidance alone in reducing musculoskeletal pain, enhancing muscle strength, and reducing analgesic use, ultimately improving employee health and reducing sick leave (119). These findings highlight the need for comprehensive ergonomic training programs, structured workplace health interventions, and systematic supervision to address knowledge gaps and reduce MSD risks among hospital cleaners.

### 4.4 Organizational factors associated with MSDs

Hospital cleaners frequently face complex and demanding work situations. However, their work is often underrecognized, characterized by low social status (47, 48). This occupation also tends to exist within unstable employment relationships (6). One study found that positive organizational factors were associated with lower physical workloads and healthier musculoskeletal conditions among hospital cleaners (51).

Several organizational issues may increase exposure to ergonomic risk factors and consequently elevate the risk of injury and illness among cleaners. These include a lack of influence or control over daily work, monotonous tasks, lack of respect or appreciation, effort-reward imbalance, insufficient supervisor or colleague support, inadequate family support, lack of training or preparation, unclear responsibilities, and poor communication (93). Therefore, comprehensive organizational improvements including enhanced job control, recognition programs, adequate training, and strengthened social support systems are essential for reducing MSD risks in this vulnerable population.

Additionally, research has indicated that task durations exceeding 2 h may expose hospital cleaners to higher MSD risks (44). Conversely, a UK study found that despite cleaners having limited autonomy in choosing cleaning tasks or rest times, reported MSD symptoms did not increase (42). However, focus group discussions in the same study emphasized that regular communication between management and cleaners plays a critical role in improving work performance and overall wellbeing (42). This discrepancy may reflect contextual differences in management practices, workload distribution, and psychosocial support across healthcare settings, highlighting that organizational communication and participatory management can buffer the physical strain associated with cleaning tasks.

### 4.5 Limitations

This systematic review has several important limitations that should be acknowledged. First, the evidence base was limited, with only 11 studies meeting the inclusion criteria, thereby limiting the comprehensiveness of our findings. The small number of studies with age-stratified data (*n* = 6) and prevalence reporting (*n* = 4) further constrained our ability to draw robust conclusions about MSDs in older hospital cleaners. Second, the literature search was restricted to English-language publications. This language limitation may have excluded relevant studies published in other languages and potentially introduced language bias. Future reviews could include non-English databases or employ translation support to reduce this limitation. Third, substantial heterogeneity existed across studies in terms of study designs, outcome measures, and assessment timeframes (7 days vs. 12 months), which precluded meta-analysis and limited comparability of findings.

Fourth, most included studies were cross-sectional in design, which only allows identification of associations rather than causal relationships between risk factors and MSDs. Regarding review process limitations, we initially set the age threshold at 55 years but adjusted it to 50 years due to insufficient studies, which may have introduced selection bias. The varying definitions of “older workers” across studies posed challenges in data synthesis. Additionally, the quality of included studies was variable, with some studies showing methodological limitations in sample selection, confounding control, and outcome measurement. Finally, self-reported outcome measures in most studies may have introduced recall bias and measurement error.

Therefore, these limitations suggest that findings should be interpreted cautiously, and future research with standardized methodologies, larger sample sizes, and longitudinal designs is needed to strengthen the evidence base for this vulnerable population.

## 5 Conclusion

This systematic review synthesized available evidence on musculoskeletal disorders among older hospital cleaners aged 50 years and above. Across 11 studies, MSD prevalence ranged from 49.1% to 68.96%, with the lower and upper back being the most affected regions. Based on the available evidence, this review proposed a preliminary conceptual model based on P-E Fit theory to understand the complex interactions between individual and environmental factors affecting MSDs in older hospital cleaners. However, further empirical research is needed to validate this theoretical framework in the future. To ensure sustainable employment for older hospital cleaners, task design and assignment require greater attention to achieve optimal person-environment fit (90). Guided by the P-E Fit theory, the findings reveal that the development of MSDs in this population results from the interaction between work demands, individual capacity, and environmental conditions.

Work demands (repetitive cleaning tasks, handling around patients, waste disposal, and prolonged standing) impose continuous physical stress on the musculoskeletal system. Individual factors, including age-related decline, gender differences, insufficient rest, psychological stress, and poor posture, further influence vulnerability to MSDs. Environmental and organizational elements, such as limited ergonomic equipment, inadequate training, unstable employment relationships, and weak managerial communication, exacerbate the imbalance between personal capacity and task requirements.

When work demands exceed individual capacity in a poorly supported environment, this imbalance increases the risk of MSDs; conversely, balance between these dimensions can reduce musculoskeletal strain and promote sustainable employability. Therefore, strategies to improve person–environment fit should focus on ergonomic equipment upgrades, task rotation, rest scheduling, targeted training, and strengthened communication between management and cleaners. Although heterogeneity among studies precluded quantitative synthesis, the findings underscore the need for integrated ergonomic, organizational, and psychosocial interventions to reduce MSD risks and promote the health of aging hospital cleaning staff. Future research with standardized methodologies and larger sample sizes is needed to strengthen the evidence base and establish more definitive risk factors for this vulnerable population.
