# Supplementary material for: Musculoskeletal disorders among older hospital cleaners: a systematic review of prevalence and risk factors
Source: Front Public Health. 2025 Nov 24;13:1711097. doi: 10.3389/fpubh.2025.1711097 (PMC12682812; doi:10.3389/fpubh.2025.1711097)
Supplement: Supplementary file 1 [file Table_1.docx]

Supplementary Material

**Supplementary Table S1**: Search strategy of databases.

| Database | Search strategy |
| --- | --- |
| PubMed | (("hospital*"[Title] OR "medical"[Title] OR "healthcare"[Title]) AND ("clean*"[Title] OR "housekeeper*"[Title] OR "janitor*"[Title] OR "custodial*"[Title] OR "environmental service*"[Title])) AND ("muscul*"[Title] OR "MSD*"[Title] OR "pain"[Title] OR "work-related injur*"[Title] OR "occupational injur*"[Title] OR "ergonomic*"[Title] OR "rheumatoid arthritis"[Title] OR "osteoarthritis"[Title] OR "gout"[Title] OR "tendinitis"[Title] OR "tenosynovitis"[Title] OR "sprain*"[Title] OR "rotator cuff injur*"[Title] OR "intervertebral disc*"[Title]) |
| MEDLINE | ("hospital*" OR "medical" OR "healthcare") (Title) AND ("clean*" OR "housekeeper*" OR "janitor*" OR "custodial*" OR "environmental service*") (Title) AND ("muscul*" OR "MSD*" OR "pain" OR "work-related injur*" OR "occupational injur*" OR "ergonomic*" OR "rheumatoid arthritis" OR "osteoarthritis" OR "gout" OR "tendinitis" OR "tenosynovitis" OR "sprain*" OR "rotator cuff injur*" OR "intervertebral disc*") (Title). |
| Web of Science | ("hospital*" OR "medical" OR "healthcare") (Title) AND ("clean*" OR "housekeeper*" OR "janitor*" OR "custodial*" OR "environmental service*") (Title) AND ("muscul*" OR "MSD*" OR "pain" OR "work-related injur*" OR "occupational injur*" OR "ergonomic*" OR "rheumatoid arthritis" OR "osteoarthritis" OR "gout" OR "tendinitis" OR "tenosynovitis" OR "sprain*" OR "rotator cuff injur*" OR "intervertebral disc*") (Title). |
| Google Scholar | ("hospital*" OR "medical" OR "healthcare") AND ("clean*" OR "housekeeper*" OR "janitor*" OR "custodial*" OR "environmental service*") AND ("muscul*" OR "MSD*" OR "pain" OR "work-related injur*" OR "occupational injur*" OR "ergonomic*" OR "rheumatoid arthritis" OR "osteoarthritis" OR "gout" OR "tendinitis" OR "tenosynovitis" OR "sprain*" OR "rotator cuff injur*" OR "intervertebral disc*"). |

**Supplementary Material S2:** PRISMA 2020 item checklist.

| Section and topic | Item # | Checklist item | Location where item is reported |
| --- | --- | --- | --- |
| **Title** |  |  |  |
| Title | 1 | Identify the report as a systematic review. | Title |
| **Abstract** |  |  |  |
| Abstract | 2 | See the PRISMA 2020 for Abstracts checklist. | Abstract |
| **Introduction** |  |  |  |
| Rationale | 3 | Describe the rationale for the review in the context of existing knowledge. | Introduction |
| Objectives | 4 | Provide an explicit statement of the objective(s) or question(s) the review addresses. | Introduction |
| **Methods** |  |  |  |
| Eligibility criteria | 5 | Specify the inclusion and exclusion criteria for the review and how studies were grouped for the syntheses. | Section 2.2 |
| Information sources | 6 | Specify all databases, registers, websites, organisations, reference lists and other sources searched or consulted to identify studies. Specify the date when each source was last searched or consulted. | Section 2.1 |
| Search strategy | 7 | Present the full search strategies for all databases, registers and websites, including any filters and limits used. | Section 2.3 |
| Selection process | 8 | Specify the methods used to decide whether a study met the inclusion criteria of the review, including how many reviewers screened each record and each report retrieved, whether they worked independently, and if applicable, details of automation tools used in the process. | Section 2.4 |
| Data collection process | 9 | Specify the methods used to collect data from reports, including how many reviewers collected data from each report, whether they worked independently, any processes for obtaining or confirming data from study investigators, and if applicable, details of automation tools used in the process. | Section 2.4 |
| Data items | 10a | List and define all outcomes for which data were sought. Specify whether all results that were compatible with each outcome domain in each study were sought (e.g. for all measures, time points, analyses), and if not, the methods used to decide which results to collect. | Section 2.5 |
|  | 10b | List and define all other variables for which data were sought (e.g. participant and intervention characteristics, funding sources). Describe any assumptions made about any missing or unclear information. | Section 2.5 |
| Study risk of bias assessment | 11 | Specify the methods used to assess risk of bias in the included studies, including details of the tool(s) used, how many reviewers assessed each study and whether they worked independently, and if applicable, details of automation tools used in the process. | Section 2.6 |
| Effect measures | 12 | Specify for each outcome the effect measure(s) (e.g. risk ratio, mean difference) used in the synthesis or presentation of results. | N/A |
| Synthesis methods | 13a | Describe the processes used to decide which studies were eligible for each synthesis (e.g. tabulating the study intervention characteristics and comparing against the planned groups for each synthesis (item #5)). | Section 2.7 |
|  | 13b | Describe any methods required to prepare the data for presentation or synthesis, such as handling of missing summary statistics, or data conversions. | N/A |
|  | 13c | Describe any methods used to tabulate or visually display results of individual studies and syntheses. | Section 2.7 |
|  | 13d | Describe any methods used to synthesise results and provide a rationale for the choice(s). If meta-analysis was performed, describe the model(s), method(s) to identify the presence and extent of statistical heterogeneity, and software package(s) used. | Section 2.7 |
|  | 13e | Describe any methods used to explore possible causes of heterogeneity among study results (e.g. subgroup analysis, meta-regression). | N/A |
|  | 13f | Describe any sensitivity analyses conducted to assess robustness of the synthesised results. | N/A |
| Reporting bias assessment | 14 | Describe any methods used to assess risk of bias due to missing results in a synthesis (arising from reporting biases). | N/A |
| Certainty assessment | 15 | Describe any methods used to assess certainty (or confidence) in the body of evidence for an outcome. | N/A |
| **Results** |  |  |  |
| Study selection | 16a | Describe the results of the search and selection process, from the number of records identified in the search to the number of studies included in the review, ideally using a flow diagram (see fig 1). | Section 3.1 |
|  | 16b | Cite studies that might appear to meet the inclusion criteria, but which were excluded, and explain why they were excluded. | Section 3.1 |
| Study characteristics | 17 | Cite each included study and present its characteristics. | Section 3.2 |
| Risk of bias in studies | 18 | Present assessments of risk of bias for each included study. | Section 3.3 |
| Results of individual studies | 19 | For all outcomes, present, for each study: (a) summary statistics for each group (where appropriate) and (b) an effect estimate and its precision (e.g. confidence/credible interval), ideally using structured tables or plots. | Section 3.4 |
| Results of syntheses | 20a | For each synthesis, briefly summarise the characteristics and risk of bias among contributing studies. | Section 3.2 |
|  | 20b | Present results of all statistical syntheses conducted. If meta-analysis was done, present for each the summary estimate and its precision (e.g. confidence/credible interval) and measures of statistical heterogeneity. If comparing groups, describe the direction of the effect. | Section 3.2 |
|  | 20c | Present results of all investigations of possible causes of heterogeneity among study results. | N/A |
|  | 20d | Present results of all sensitivity analyses conducted to assess the robustness of the synthesised results. | N/A |
| Reporting biases | 21 | Present assessments of risk of bias due to missing results (arising from reporting biases) for each synthesis assessed. | Section 3.3 |
| Certainty of evidence | 22 | Present assessments of certainty (or confidence) in the body of evidence for each outcome assessed. | Section 3.3 |
| **Discussion** |  |  |  |
| Discussion | 23a | Provide a general interpretation of the results in the context of other evidence. | Discussion |
|  | 23b | Discuss any limitations of the evidence included in the review. | Section 4.6 |
|  | 23c | Discuss any limitations of the review processes used. | Section 4.6 |
|  | 23d | Discuss implications of the results for practice, policy, and future research. | Section 4.2; Section 4.3;  Section 4.4;  Section 4.5 |
| **Other information** | |  |  |
| Registration and protocol | 24a | Provide registration information for the review, including register name and registration number, or state that the review was not registered. | Methods |
|  | 24b | Indicate where the review protocol can be accessed, or state that a protocol was not prepared. | Methods |
|  | 24c | Describe and explain any amendments to information provided at registration or in the protocol. | Methods |
| Support | 25 | Describe sources of financial or non-financial support for the review, and the role of the funders or sponsors in the review. | Funding |
| Competing interests | 26 | Declare any competing interests of review authors. | Declarations of interest |
| Availability of data, code, and other materials | 27 | Report which of the following are publicly available and where they can be found: template data collection forms; data extracted from included studies; data used for all analyses; analytic code; any other materials used in the review. | Data Availability Statement |

**Table 1.** Six systematic reviews on MSDs among cleaners.

| **Title** | **Year** | **Population** | **Research questions** | **N** | **Key findings** |
| --- | --- | --- | --- | --- | --- |
| Prevalence of musculoskeletal disorders among hotel housekeepers and cleaners: A systematic review with meta-analysis | 2024 | Hotel housekeepers and cleaners | What is the prevalence of MSD among hotel housekeepers and cleaners? | 28 | Musculoskeletal disorders are highly prevalent among hotel housekeepers and cleaners; Low back pain is the most common MSD; The most affected anatomical locations: low back, shoulders, and wrists/hands. |
| Musculoskeletal risk factors in cleaning occupation – A literature review | 2008 | Cleaners | What are the musculoskeletal risk factors in cleaning occupations? | - | Cleaning work is associated with high physical and psychosocial workloads. Significant research gaps exist in: facility design for cleanability; ergonomic design of cleaning tools; individual risk factors; limited job control and career development. |
| Occupational hazards experienced by cleaning workers and janitors: A review of the epidemiologic literature | 2009 | Cleaning workers and janitor | What are the occupational health hazards and associated health outcomes experienced by cleaning workers and janitors based on epidemiologic evidence? | 35 | Five major health outcome categories were identified: respiratory diseases, dermatologic disorders, infectious diseases, musculoskeletal disorders, and mental disorders. |
| Cleaning in the 21st Century: The musculoskeletal disorders associated with the centuries-old occupation-A literature review | 2022 | Cleaners | What are the current trends and factors contributing to MSDs in cleaning workers? | 39 | Higher MSD risk in the cleaning industry; Task factors and individual factors received most research attention, while interaction elements were severely under-studied; MSD causation is multifaceted. |
| Occupational health hazards of street cleaners-a literature review considering prevention practices at the workplace | 2020 | Street cleaners | What are the occupational health hazards faced by street cleaners globally, and what prevention practices can be implemented at the workplace? | 45 | Proper education on hazard awareness and supportive workplace communication are essential to reduce both physical and mental stress in street cleaners. |
| Strategies to minimize ergonomic risks in the cleaning staff: an integrative review | 2021 | Cleaning staff | What strategies can effectively minimize ergonomic risks among cleaning workers? | 21 | Primary ergonomic education strategy, supplemented by aerobic exercise; Use equipment with better ergonomic design and inter-functional layout; Prioritize optimized and easily adjustable mop handles. |

**Table 2.** Characteristics of included studies.

| **No.** | **Country** | **Design** | **Study Period** | **Setting** | **N (Female %)** | **Age** | **Primary Outcome** | **MSD** assessment method | **Risk Factors** | **Prevalence (%)** | **Sample Selection** | **Confounding Control** | **Limitations** |
| --- | --- | --- | --- | --- | --- | --- | --- | --- | --- | --- | --- | --- | --- |
| (25) | Brazil | Cross-sectional | 2024 | Public  hospital | 149 (89.9) | 23-62 | Work-related MSP prevalence | The Nordic musculoskeletal questionnaire | Staffing dissatisfaction; Alcohol use; Self-medication; Sedentary lifestyle; Smoking; Medication use; Self-medication; Married status; Alcohol use; Sedentary lifestyle; Sleep <8h/day | Lower back: 42.3%; Ankle/Foot: 28.2%; Wrist/Hand: 27.5%; Upper back: 25.5%; Shoulder: 23.5% | Convenience sampling | N/A | The cross-sectional study design precludes establishing temporality between exposure and outcome. |
| (26) | France | Cross-sectional | 2024 | Public hospital | 8 (12.5) | 38.67± 13.64 | Ergonomic Scores | Nordic musculoskeletal questionnaire; [rapid upper limb assessment](https://www.mdpi.com/search?q=rapid+upper+limb+assessment) | Move operating tables, stretchers, and carts | Neck (66.7%);  Shoulders (66.7%);  Elbows (11.1%);  Wrists (44.4%);  Upper back (44.4%); Lower back (77.8%) | Convenience sampling | N/A | Unable to collect all the necessary information required for the RULA ergonomic assessment; sample size is insufficient; There is no consensus on the definition of the sub-tasks; Lack of epidemiological data to support the proposed model. |
| (32) | Sweden | Prospective cohort | 2001 | Public healthcare sector | 45 (100) | 27-62 | Prevalence of MSDs in female workers continuing to work despite symptoms | Pain screening questionnaire; Visual analogue scale (VAS) | N/A | Shoulder girdle: 51.1%; Low back: 22.2%; Hip muscles: 22.2%; Rotator cuff: 17.8%; Neck muscles: 15.6% | Convenience Sampling | Limited (Design-based matching only, no statistical adjustment) | Non-randomized design; Small sample; Unblinded evaluation; Limited statistical adjustment |
| (33) | Brazil | Cross-sectional | 2017 | Public university hospital | 157 (87.9) | 39.9±9.78 | prevalence and factors associated with the occurrence of musculoskeletal pain | VAS (0-10); Semi-structured interview | Age (younger workers 19-34 years); Limited leisure time | Age: 19-34 (76.5%); 35-44 (78%); 45-60 (57.1%) | Census Sampling | Statistical adjustment using Poisson regression; Multivariable models controlling for age, leisure time, and other confounding variables | Cross-sectional design; Small sample size; Limited statistical power for occupational factors |
| (6) | Thailand | Cross-sectional | 2021 | Public hospital | 331 (82.2) | 20-63 | the prevalence of musculoskeletal disorders (MSDs) and related factors | The stress test questionnaire; The work characteristics questionnaire; The standardized Nordic questionnaire | Male gender; Severe stress; History of injuries; Mopping posture and task duration for 2–4 and for more than 4 h. | seven-day (73.9%) and 12-month (81.9%), the lower back (57.7%), followed by the shoulder (52.6%). | Census Sampling | N/A | Cross-sectional study; Recall bias; Generalizability limited to similar contexts |
| (27) | Peru | Cross-sectional | 2020 | Public hospital | 129 (82.9) | Median = 43; IQR = 15 | the prevalence of musculoskeletal disorders (MSDs) and related factors | The Standard Nordic Questionnaire (SNQ) | No factors associated with musculoskeletal disorders were found | 93.02%; lumbar pain (65.1%), dorsal back pain (47.3%), neck pain (37.2%), and elbow/forearm pain (13.2%). | Census Sampling | Statistical adjustment using logistic regression; Limited multivariable control for age, work time, BMI | Cross-sectional design prevents causal inference; Insufficient statistical power due to limited sample size; Single hospital setting limits generalizability |
| (29) | USA | Case-control | 2007 | N/A | 664 (72) | 45±10 | The combined impact of economic, organizational, psychosocial and physical factors on work-related musculoskeletal disorders | Structured questionnaire; The ergonomics assessments | Occupation (other clinical workers); Effort-reward imbalance; Ergonomic exposures (lower body strain) | N/A | Convenience sampling | Multivariate logistic regression with matching (job group, shift length); Hospital site adjustment; Systematic variable selection (P<0.20) | Cross-sectional; Self-report bias; Limited generalizability; No baseline data |
| (30) | USA | Prospective cohort | 2019 | N/A | 390 (55) | 18-30 (10%); 31-40 (30%);41-50 (30%);51-60 (23%); ≥60 (6%) | To assess if the ergonomic workload is related to [injuries](https://www.sciencedirect.com/topics/pharmacology-toxicology-and-pharmaceutical-science/injury) among janitors. | Self-reported questionnaire; Ergonomic assessment tools (REBA and Borg scale) | Heavy ergonomic workload | N/A | Convenience sampling | Multivariable log-binomial regression adjusted for age, gender, smoking status, job title; Inverse probability weighting for response bias; Generalized Estimating Equations (GEE) for clustering bias | Low response rate (32.5%); Laboratory simulation generalizability issues; Observer angle judgment accuracy limitations; Potential incidence-prevalence bias; Injury underreporting due to fear of reprisal; Language limitations and distrust affecting response rate |
| (31) | Turkey | Cross-sectional | 2021 | Public hospital | 137 (82.5) | 26-35 (59.8%); ≤25 (26.3%); ≥36(13.9%) | Conduct a preventive assessment of whether the working environment and furniture design comply with ergonomic principles | Self-reported questionnaire on workplace furniture ergonomics and satisfaction | Workplace furniture ergonomic factors and environmental conditions | N/A | Convenience sampling | N/A | Single hospital study; convenience sampling; cross-sectional design |
| (2) | UK | Cross-sectional | 2006 | Public hospital | 1216 (89) | 49±12.5 | To investigate musculoskeletal health among UK cleaners and provide organizational recommendations to reduce workplace injuries | Self-reported questionnaire surveys; Workplace observational assessments; Interviews | Manual handling, awkward postures, equipment factors, work organization factors | 74%; Low back (46%); neck (33%); knees (24%); right shoulder (23%); right wrist/hand (22%) | Mixed sampling: Random sampling; Convenience sampling; Purposive sampling | N/A | Small sample size; Low questionnaire response rate; Self-reported data with potential recall bias; Language barriers affecting participation; Cross-sectional design; Possible selection bias |
| (28) | USA | Cross-sectional | 2011 | N/A | 106 (-) | 19-72 | To determine the prevalence of musculoskeletal disorders in hospital cleaners | An interview-based modified Nordic Questionnaire | slip/trip/fall and material handling | 64.2%;  Lower back (49%); Wrists (43% right, 35% left); Knees (34% right, 35% left); Shoulders (25% right, 21% left) | Convenience sampling | N/A | Non-generalizable findings; Self-reported data; Recall bias; Lack of validation through workers' compensation claims data |

**Table 3.** The JBI checklist to evaluate the risk of bias of included studies.

| **No.** | **Included**  **Study** | **Q1** | **Q2** | **Q3** | **Q4** | **Q5** | **Q6** | **Q7** | **Q8** | **Total** | | **Quality** | |
| --- | --- | --- | --- | --- | --- | --- | --- | --- | --- | --- | --- | --- | --- |
| 1 | (25) | Y | Y | Y | Y | Y | N | Y | Y | | 7/8 (87.5%) | | High |
| 2 | (26) | Y | Y | Y | Y | U | N | Y | Y | | 6/8 (75%) | | Moderate |
| 3 | (32) | U | Y | Y | Y | Y | U | Y | Y | | 6/8 (75%) | | Moderate |
| 4 | (33) | Y | Y | Y | Y | Y | Y | Y | Y | | 8/8 (100%) | | High |
| 5 | (6) | Y | Y | Y | Y | Y | Y | Y | Y | | 8/8 (100%) | | High |
| 6 | (27) | Y | Y | Y | Y | Y | Y | Y | N | | 7/8 (87.5%) | | High |
| 7 | (29) | Y | Y | Y | Y | Y | Y | Y | Y | | 8/8 (100%) | | High |
| 8 | (30) | Y | Y | Y | Y | Y | Y | Y | Y | | 8/8 (100%) | | High |
| 9 | (31) | Y | Y | Y | Y | U | N | Y | Y | | 6/8 (75%) | | Moderate |
| 10 | (2) | Y | Y | Y | Y | U | N | Y | Y | | 6/8 (75%) | | Moderate |
| 11 | (28) | U | Y | Y | Y | Y | U | Y | U | | 5/8 (62.5%) | | Moderate |

Y: Yes; N: No; U: Unclear.

**Table 4.** the prevalence of MSDs among older hospital cleaners.

| **Study** | **Age group** | **Period** | **Prevalence** |
| --- | --- | --- | --- |
| (25) | 49-62 years | The last 7days | Neck (13.5%); shoulders (17.3%); elbows (25%); wrists/hands (23.1%); upper back (42.3%); lower back (42.3%); hip/thighs (30.8%); knees (34.6%); ankles/feet (21.2%) |
| (28) | 39-58 years;  >59 years | The last 12 months | 68.96%;  66.66% |
| (33) | 45-60 years | The last 7days | 57.1% |
| (6) | 40-63 years | The last 12 months | 49.1% |
